# PosturePose: Optimized Posture Analysis for Semi-Supervised Monocular 3D Human Pose Estimation

**DOI:** 10.3390/s23249749

**Published:** 2023-12-11

**Authors:** Lawrence Amadi, Gady Agam

**Affiliations:** Visual Computing Lab, Illinois Institute of Technology, Chicago, IL 60616, USA; agam@iit.edu

**Keywords:** human posture analysis, human pose estimation, semi-supervised pose estimation, weakly supervised pose estimation

## Abstract

One motivation for studying semi-supervised techniques for human pose estimation is to compensate for the lack of variety in curated 3D human pose datasets by combining labeled 3D pose data with readily available unlabeled video data—effectively, leveraging the annotations of the former and the rich variety of the latter to train more robust pose estimators. In this paper, we propose a novel, fully differentiable posture consistency loss that is unaffected by camera orientation and improves monocular human pose estimators trained with limited labeled 3D pose data. Our semi-supervised monocular 3D pose framework combines biomechanical pose regularization with a multi-view posture (and pose) consistency objective function. We show that posture optimization was effective at decreasing pose estimation errors when applied to a 2D–3D lifting network (VPose3D) and two well-studied datasets (H36M and 3DHP). Specifically, the proposed semi-supervised framework with multi-view posture and pose loss lowered the mean per-joint position error (MPJPE) of leading semi-supervised methods by up to 15% (−7.6 mm) when camera parameters of unlabeled poses were provided. Without camera parameters, our semi-supervised framework with posture loss improved semi-supervised state-of-the-art methods by 17% (−15.6 mm decrease in MPJPE). Overall, our pose models compete favorably with other high-performing pose models trained under similar conditions with limited labeled data.

## 1. Introduction

A major challenge for 3D human pose estimation (HPE) in the wild is acquiring unconstrained training data annotated with accurate 3D poses. Human pose datasets used to train and evaluate 3D pose estimators [1,2,3,4,5] typically contain a few million poses of a limited set of persons, activities, and scenery. For example, the most studied H36M dataset [1] has 1.5 M poses in its training set, with only five persons performing 15 distinct actions in the same room. Although a greater variety of subjects performing various activities in different environments is needed to train robust pose estimators, it is costly and difficult to curate such a rich dataset because of the constraining setup of synchronized cameras and motion tracking systems needed to generate accurate joint annotations. This inadequate variety in training data is one reason why leading pose estimators struggle to replicate their high performance when tested in the wild, especially when exposed to unfamiliar activities and environments. Our objective was to study semi-supervised training techniques that would allow us to leverage the annotations of curated datasets and the richness of unconstrained unlabeled video data to fine-tune state-of-the-art (SOTA) pose estimators. This paper presents a semi-supervised training pipeline for monocular 3D pose estimation that learns from both labeled and unlabeled data by (1) constraining the biomechanical and kinematic properties of unlabeled poses using pose prior regularization [6] and (2) optimizing the multi-view pose and posture consistency of estimated 3D poses. We present a fully differentiable bone alignment procedure, which is the basis of our proposed posture loss term and evaluation metrics. Differentiability enables the seamless propagation of posture error through the bone alignment procedure to update the network parameters responsible for estimating the 3D pose, without leaving the network’s computation graph. This results in the faster execution of backpropagation and optimization operations during training and the faster and more accurate convergence of the network, as the gradients of the posture loss are retained through the bone alignment procedure. We make the following contributions:

1. We propose a new posture metric that assesses the similarity between poses by comparing the relative orientation of bones irrespective of the poses’ global positioning and orientation. Unlike existing normalized pose evaluation protocols, our posture metric is better at isolating errors to the defaulting joints and bones.
2. The proposed posture metric is fully differentiable and therefore can be directly optimized. We demonstrate its efficacy as a multi-view posture consistency loss function that can be jointly optimized with multi-view pose consistency loss on unlabeled poses in a weakly supervised training pipeline. The addition of these loss terms significantly improves upon monocular pose estimators.
3. We present a posture-centric semi-supervised scheme for pose estimation that does not require intrinsic or extrinsic camera parameters and no 2D or 3D pose annotations for the majority of the training data. We significantly improve SOTA semi-supervised pose estimation performance without camera parameter annotations (i.e., no ground truth or estimated camera parameters are used).

## 2. Related Work

We review existing 3D-HPE methods at varying degrees of supervision.

Fully Supervised 3D-HPE with 3D Pose Ground Truth

The full supervision of deep learning models involves direct optimization by comparing their predicted output to the expected ground truth. Pose estimation methods in this category learn a mapping from 2D to 3D poses by supervising pairs of 2D–3D correspondence. Fully supervised multi-view 3D pose estimators [7,8,9,10,11] lead pose estimation accuracy with 17.6 mm mean per-joint position error (MPJPE) state-of-the-art performance on H36M [12]. However, most real-world scenarios are restricted to a single viewpoint. This motivates the study of monocular (single-view) 3D human pose estimation. The leading monocular 3D-HPE networks are trained with full supervision [13,14,15,16,17,18] and have obtained a mean joint position error as low as 21.6 mm on the H36M dataset. We believe that more training data with richer variety is key to bridging the performance gap between monocular and multi-view 3D-HPE. However, curating a large dataset with a rich variety of persons, activities, and scenery is difficult to accomplish because of the non-trivial setup of motion capture systems used to generate 3D pose annotations. As an alternative, some works have proposed the use of additional synthetic training data generated by stitching together image patches [19,20,21] or poses [22], using graphics engines [23,24], or directly augmenting 2D and 3D pose pairs via a jointly trained GAN that learns to generate realistic 3D poses [25,26,27]. Other works have explored weakly and semi-supervised 3D-HPE to distill knowledge from large amounts of unlabeled data and leverage its rich pose variety.

Weakly Supervised 3D-HPE without 3D Pose Ground Truth

The concept of the weak or self-supervision of deep neural networks involves the implicit optimization of a model either without the knowledge of the expected output or without a direct comparison between each predicted output and the corresponding ground-truth target. The appeal of studying self- or weakly supervised deep learning techniques is the lower reliance on the availability of structured annotated training data, as they can be difficult to obtain in large amounts for some deep learning problems. Pose estimation works in this category include methods designed to train pose estimators without 3D pose annotations [28,29,30,31] or use 3D pose annotations to train a network without one-to-one correspondence between input 2D images or poses and the target 3D pose annotations [32,33]. Zhou et al. [33] augmented a 2D pose estimator with a depth regression sub-network and jointly trained both sub-nets with 2D and 3D labels to fully exploit the correlation between 2D pose and depth estimation sub-tasks. Other works like [34] looked to exploit multi-view information only during training. Hua et al. [35] proposed a U-shaped cross-view graph convolution network (GCN) that was trained without 3D labels. Instead, a triangulation and refinement procedure was performed across two views to lift 2D keypoints into coarse 3D poses. Iqbal et al. [36] presented a weakly supervised framework that optimized multi-view consistency. Given 2D images, their network estimated 2.5D poses (2D joint heat maps and depth maps) from which scale-normalized 3D poses were reconstructed. However, unlike our method, their multi-view consistency loss relied on a non-differentiable rigid alignment procedure and intrinsic camera parameters. Wandt et al. (CanonPose) [37] proposed a self-supervised method that exploited the multi-view constraint by projecting the estimated 3D pose in one view to a 2D pose in another view and optimizing juxtaposed reprojected 2D losses. The following self-supervised works proposed different strategies for acquiring 3D pose annotations from multi-view 2D data. Gholami et al. (TriPose) [38] triangulated a 3D pose given 2D poses from multiple views and estimated the relative orientation of poses. The triangulated 3D poses were then used as pseudo-annotations to train their 2D–3D pose lifting network. Kocabas et al. (EpipolarPose) [39] presented a self-supervised method that utilized Epipolar geometry to obtain person and camera 3D poses from multi-view 2D images that were used as pseudo-labels to train their pose network.

Semi-Supervised 3D-HPE with some 3D Pose Ground Truth

Semi-supervised deep learning techniques look to leverage the advantages of full and weak supervision by training a model on a structured subset of annotated data and a larger subset of unlabeled data rich in variety. Thus, the model converges faster to a stable optimum because of the full supervision while attaining generalizability robustness thanks to the improved distribution of unlabeled data used in weak supervision. This category of 3D-HPE works tries to learn more robust 3D pose estimators by combining annotated 3D pose training data with much more unlabeled video data. Existing works [40,41,42] have employed a dual-branch training pipeline with a fully supervised branch and a self-supervised branch that learns from the 2D pose inputs without 3D pose annotations. Rhodin et al. [43] proposed addressing the problem of insufficiently large training samples by learning a latent representation of 3D geometry from multi-view 2D images. Wang et al. [44] trained a 3D pose estimator by distilling knowledge from a modified non-rigid structure from motion (NRSfM) network used to reconstruct 3D shapes and camera positions from multiple 2D poses. To reduce over-reliance on the reprojected 2D loss, some works have employed adversarial networks that learn a distribution of realistic poses [25,45]. Gong et al. [25] presented an auto-augmentation GAN framework that learned to generate realistic 2D–3D poses, thereby increasing the quantity and diversity of supervised training data. Other works have focused on enforcing kinematic and pose geometry constraints on semi-supervised 3D pose encodings [33,46,47,48,49]. Amadi and Agam [6] proposed two effective biomechanical pose prior regularizers—bone proportion and joint mobility constraints—introduced to the weakly supervised branch to regulate overfitting to the 2D reprojection loss and directly optimize plausible 3D poses.

In the Context of Our Work

Naively enforcing multi-view consistency can lead to degenerated solutions. For example, triangulating 3D poses from estimated multi-view 2D poses using bundle adjustment may produce inaccurate results, especially when employing estimated camera parameters. Consequently, supervising the network with sub-optimal triangulated 3D poses may adversely affect performance. Previous works either used partial 3D annotation [50], learned a multi-view latent embedding of 3D poses [51], proposed a 2.5D approach to constrain the solution space [36], or used multi-view projection loss [37]. Iqbal et al. [36] used Procrustes analysis to derive a rotation matrix that aligns reconstructed multi-view 3D poses before computing the joint position error loss. However, Procrustes alignment is non-differentiable because it involves singular-value decomposition. Therefore, the crucial computation of the rotation matrix must be detached from the network’s computation graph. This implies that the network cannot backpropagate through the derivation of the rotation matrix, which directly influences the loss being optimized. Unlike Iqbal et al., our proposed method is end-to-end because our posture loss uses a novel differentiable bone orientation alignment. This allows the resulting loss to be backpropagated through the alignment protocol, thereby maximizing network optimization at each training iteration. Our work falls under the category of monocular semi-supervised 3D-HPE. Our proposed multi-view posture consistency loss is a soft constraint that teaches the network to learn consistent 3D pose encoding across multiple viewpoints. We propose a framework that optimizes multi-view pose and posture consistency without 2D and 3D pose annotations for the majority of training data while utilizing biomechanical pose regularization techniques [6] to constrain the 3D pose geometric properties. We also present a modified semi-supervised framework that does not require camera parameters.

## 3. Method

Reconstructed 3D pose error is typically the main optimization objective of pose estimation networks. Most pose models learn by minimizing the L2-norm between joints or the **mean per-joint position error (MPJPE)**, which computes the Euclidean distance between estimated and ground-truth joints after aligning the root joints (typically the pelvis) of both poses. The L2-norm optimizes the 3D pose and location in space with respect to the observing camera, while the MPJPE optimizes the 3D pose irrespective of global placement. We decouple a 3D pose into global placement, orientation, and 3D posture, where posture captures the positioning of each joint relative to other joints, and independent of the camera bearings. Hence, posture is invariant to the global placement and orientation of the pose with respect to the observing camera. By extracting posture from pose we can analyze the structural correctness of 3D poses beyond the confines of the camera position and orientation. This becomes especially useful when exploiting multi-view information to train pose estimators without 3D joint and camera parameter annotations.

In the following sections, we first review the concept of 3D pose prior regularization adopted from an existing work to constrain the biomechanical properties of estimated 3D poses. We then describe the bone alignment procedure that is critical to extracting posture and is unaffected by the positioning, scale, and orientation of 3D poses in Section 3.2. We then formulate the posture loss (Section 3.3) and evaluation metrics (Section 3.4) and elaborate on the peculiar attributes that distinguish them from existing pose estimation objectives and evaluation protocols. We describe the semi-supervised schemes used to train pose estimator networks, first with camera parameters (Section 3.3.1) and then without camera parameters (Section 3.3.2). The resulting performance of these configurations is discussed in Section 4.

### 3.1. Biomechanical Pose Prior Regularization

This work builds upon our existing work on modeling the innate bone proportion and joint mobility properties of 3D human poses and constraining these biomechanical properties when training a weakly supervised 3D pose estimation network. The semi-supervised frameworks we propose in this work are bootstrapped by the biomechanical pose prior regularizers introduced by Amadi and Agam [6]. Here, we briefly summarize the pose prior regularizers but refer the reader to the cited work for more details.

**Figure 1:**
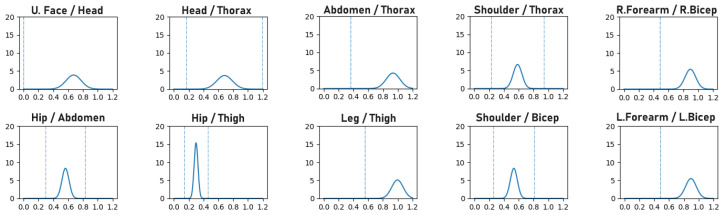
The line plots represent the likelihood (Y-axis) of bone proportion values (X-axis) for pairs of bone ratios. For example, “Hip/Thigh” is the ratio of the length of the hip and thigh bones. The vertical dotted lines indicate the range of values for each pair of bones observed in the training data.

**Figure 2:**
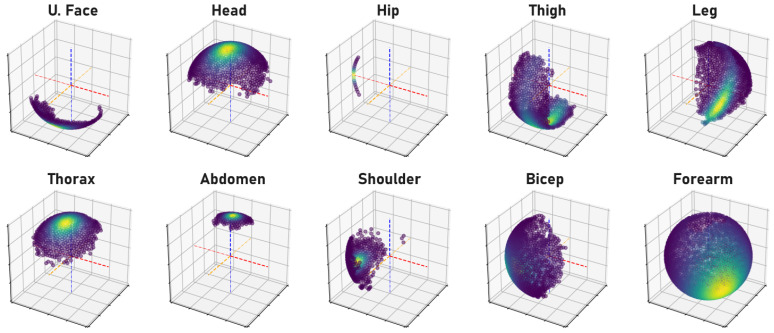
The point-cloud plots are visual representations of the likelihood of plausible orientations of each bone. The orientation of a bone is captured by the bone’s unit vector after alignment, with one end of the vector rooted to the Cartesian origin while the other end rests on the spherical surface. Bright to dark colored regions on the spherical surface indicate high to low likelihoods of orientations observed in the training data. Symmetrical parts like the right and left shoulder are grouped together. The red, blue, and orange lines are the XYZ axis, respectively.

Human bone proportions and joint rotations are modeled by observing the annotated 3D pose training data of the H36M dataset to compute the mean and variance of the probability density functions (PDFs) that model the likelihood of bone proportions and orientations, as illustrated in Figure 1 and Figure 2, respectively. When training a network, the precomputed PDFs are used to assess the likelihood of the bone proportions and orientations of an estimated 3D pose. The model learns better pose estimation by maximizing the log-likelihood of bone proportions and joint rotations. By maximizing the log-likelihood of these biomechanical properties, the network is forced to encode more correct 3D poses.

### 3.2. Differentiable Bone Orientation Alignment

**Figure 3:**
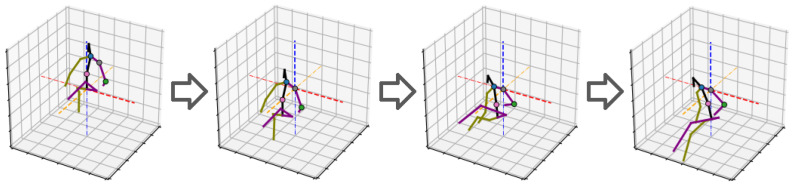
A step-by-step illustration of the left bicep bone orientation alignment with the left shoulder and elbow as the Pivot (grey dot) and Free (green dot) keypoints, and the neck and spine as the Axis (blue dot) and Anchor (pink dot) keypoints. From left to right, the pose in the 1st image is translated to place the Pivot keypoint at the origin. Then, it is rotated so that the Axis-Bone aligns with the X-axis in the 3rd image. Finally, the pose is rotated so the Anchor-Plane aligns with the XY-Plane. The Free-Bone vector in the 4th image describes the orientation of the left bicep. The red, blue, and orange lines are the XYZ axis. This procedure is vital to extract the true orientation and posture of a bone independent of the orientation, position, and scale of the 3D pose.

The objective of the proposed bone alignment procedure is to transform components of 3D poses in a standardized manner that facilitates the retrieval of the true orientation of each bone. The goal is to extract the orientation of each bone relative to other neighboring bones, irrespective of the size, global positioning, and general orientation of the 3D poses. This is achieved by selecting a set of four joints in close proximity to each other to guide the alignment of a bone. We start with the **Pivot** and **Free** keypoints, which are the joints at either end of the bone whose true orientation we want to extract. The other two joints are the **Axis** and **Anchor** keypoints. The Axis keypoint forms the **Axis-Bone** with the Pivot keypoint, and the Pivot, Axis, and Anchor keypoints define a distinct plane that we refer to as the **Anchor-Plane**. The purpose of the alignment procedure is to align the Anchor plane to the Cartesian XY-Plane with the Pivot keypoint at the origin and the Axis-Bone aligned with the X (or Y) axis. The outcome of this transformation is that the orientation of the Free-Bone is normalized with respect to the Axis and Anchor keypoints in a way that is invariant to the translation and rotation of the entire 3D pose. We ensure invariance to scale by extracting the Free-Bone’s unit vector after alignment. This alignment transformation is carried out for each bone with their corresponding, hand-selected, quadruplet keypoints. The procedure is illustrated for clarity in Figure 3 with the example of right elbow alignment and described mathematically below.

Given each set of quadruplet keypoints $Q_{f}^{p}$ of a 3D pose *P*, the goal is to align the Pivot, Axis, and Anchor keypoints with the XY-Plane. Note that $Q_{f}^{p}=\left\{j_{f},j_{p},j_{a},j_{c}\right\}$ contains the 3D coordinates of the Free, Pivot, Axis, and Anchor keypoints, respectively. We first translate $Q_{f}^{p}$ so that the Pivot keypoint $j_{p}$ moves to the Cartesian origin.

(1) $$Q_{f}^{p^{\prime }}=Q_{f}^{p}-j_{p}=\left\{j_{f}^{{}^{\prime }},j_{p}^{{}^{\prime }},j_{a}^{{}^{\prime }},j_{c}^{{}^{\prime }}\right\}\;$$

Next, we build a rotation matrix $R_{f}^{p}=\left[{\hat{u}}_{f}^{i},{\hat{u}}_{f}^{j},{\hat{u}}_{f}^{k}\right]$ to rotate the Free-Bone vector. The unit vectors corresponding to the X-, Y-, and Z-axis of the rotation matrix are derived below.

(2) $${\vec{v}}_{f}^{k}=j_{a}^{{}^{\prime }}\times j_{c}^{{}^{\prime }}\;or\;j_{c}^{{}^{\prime }}\times j_{a}^{{}^{\prime }}$$

(3) $$\begin{array}{cc} j^{{}^{\prime }} & =j_{a}^{{}^{\prime }}\;or\;j_{c}^{{}^{\prime }}\\ {\vec{v}}_{f}^{c} & ={\vec{v}}_{f}^{k}\times j^{{}^{\prime }}\;or\;j^{{}^{\prime }}\times {\vec{v}}_{f}^{k} \end{array}\;$$

(4) $${\hat{u}}_{f}^{a}=\delta_{f}^{a}\cdot \frac{j_{a}^{{}^{\prime }}}{|j_{a}^{{}^{\prime }}|},\;{\hat{u}}_{f}^{c}=\delta_{f}^{c}\cdot \frac{{\vec{v}}_{f}^{c}}{|{\vec{v}}_{f}^{c}|},\;{\hat{u}}_{f}^{k}=\frac{{\vec{v}}_{f}^{k}}{|{\vec{v}}_{f}^{k}|}$$

where $\delta_{f}^{a},\delta_{f}^{c}\in \left\{-1,1\right\}$ changes the direction of the unit vector. Note that the order of the cross products in Equations (2) and (3) and the choice of $\delta_{f}^{a},\delta_{f}^{c}$ and $j^{{}^{\prime }}$ for each bone alignment are guided by the right-hand rule and the relative positioning of the quadruplet keypoints with respect to the structure of a standard skeletal pose. The selected configurations for all 16 bones are provided in the Supplementary Materials. The intuition behind the derivation of the rotation matrix is that the Axis-Bone defines the direction of the new X- (or Y-) axis. The normal vector to the Anchor-Plane is the direction of the new Z-axis and the orthogonal vector between the new Z-axis, and the Axis-Bone defines the direction of the new Y- (or X-) axis. Note that the Axis-Bone may be horizontally aligned with the X-axis or vertically aligned with the Y-axis, depending on the Free-Bone. When horizontally aligned, the superscripts *a* = *i* and *c* = *j* in Equations (3) and (4) (i.e., ${\hat{u}}_{f}^{i}$ = ${\hat{u}}_{f}^{a}$, ${\hat{u}}_{f}^{j}$ = ${\hat{u}}_{f}^{c}$). Otherwise, when vertically aligned, *c* = *i* and *a* = *j* (i.e., ${\hat{u}}_{f}^{i}$ = ${\hat{u}}_{f}^{c}$, ${\hat{u}}_{f}^{j}$ = ${\hat{u}}_{f}^{a}$). Finally, the orientation of the Free-Bone ${\hat{b}}_{f}$ is extracted after rotation alignment in Equation (5). $b_{f}^{\left(h\right)}$ and $b_{f}^{\left(x,y,z\right)}$ are the homogeneous and (x,y,z) components of the rotated bone, respectively.

(5) $$\begin{array}{ccc} b_{f} & =\left[\begin{array}{cc} R_{f}^{p} & 0\\ 0 & 1 \end{array}\right]\left[\begin{array}{c} j_{f}^{{}^{\prime }}\\ 1 \end{array}\right]\\ {\vec{b}}_{f} & =\frac{b_{f}^{\left(x,y,z\right)}}{b_{f}^{\left(h\right)}}\;and\;{\hat{b}}_{f} & =\frac{{\vec{b}}_{f}}{|{\vec{b}}_{f}|} \end{array}\;$$

This alignment procedure can be implemented such that the computations are vectorized as tensor operations and executed at once for all poses and bones in a batch. This makes it a fast and memory-efficient procedure. Our preset configurations for executing bone orientation alignment for each bone and the resulting 3D pose transformation effect are presented in Appendix B.

### 3.3. Bone Orientation Error for Posture Loss

Following the Free-Bone alignment procedure, we can easily assess the dissimilarity between the isolated orientation of pairs of bones (i.e., the same bone in any two given 3D poses) by computing the distance between their aligned Free-Bone unit vectors. These can be pairs of bones in 3D poses estimated from different viewpoints or pairs of bones in an estimated 3D pose and the corresponding ground-truth 3D pose. This gives us a measure of the orientation of each pair of bones invariant to the global orientation, positioning, and scale of either 3D pose. Collectively, we can evaluate the posture similarity between pairs of 3D poses. Unlike the rigid alignment of the **Procrustes mean per-joint position error (P-MPJPE)** that leads to numerical instabilities during backpropagation due to singular-value decomposition, our Free-Bone alignment procedure is fully differentiable as it involves basic addition, subtraction, multiplication, and division operations. This advantage over the P-MPJPE enables the direct optimization of 3D posture as a loss term when training a pose estimator. Our proposed **mean per-bone orientation error (MPBOE)** for a batch of poses *P* and set of bones *B* is defined in Equation (6).

(6) $$L_{\mathrm{posture}}=\frac{1}{\left|P||B\right|}\sum_{P}\sum_{f\in B}d\left(\alpha_{f}{\hat{b}}_{f},\alpha_{f}{\hat{b}}_{f}^{{}^{\prime }}\right)\;$$

where *d* is a distance measure (e.g., L2-norm, L1-norm, or cosine similarity); ${\hat{b}}_{f}$ is the Free-Bone unit vector of the estimated 3D pose after alignment; and ${\hat{b}}_{f}^{{}^{\prime }}$ is the corresponding Free-Bone unit vector of the ground-truth 3D pose after alignment. Both unit vectors are scaled by $\alpha_{f}$, which is the length of the corresponding ground-truth bone. This normalization is critical to distribute the weight of the posture loss term amongst the bones of a 3D pose such that the influence of a bone’s orientation error is directly proportional to the length of the bone. Otherwise, shorter and more rigid torso bones would have the same influence as longer and more agile limb bones, resulting in poorer performance. This posture loss can be jointly optimized with the MPJPE as an auxiliary loss term in a fully supervised setting. It can also be minimized for weakly supervised multi-view poses in a semi-supervised setting as in Equation (7).

(7) $$L_{\mathrm{posture}}^{{}^{\prime }}=\frac{1}{m|P||B|}\sum_{P}\sum_{f\in B}\sum_{c=0}^{m}d\left(\alpha_{f}{\hat{b}}_{f}^{c},\alpha_{f}{\hat{b}}_{f}^{c+1}\right)\;$$

Given a set of cameras *C*, m=|C|−1, ${\hat{b}}_{f}^{c}$ is the Free-Bone-aligned unit vector of the pose estimated for the viewpoint of camera *c*. Note that in a semi-supervised setting, there are no ground-truth 3D poses in the weakly supervised branch. $\alpha_{f}$ is computed from the batch of annotated 3D poses in the fully supervised branch as the mean bone length of the corresponding bone. We set *d* as the L1-norm between vectors.

#### 3.3.1. Semi-Supervision with Multi-View Posture Loss

**Figure 4:**
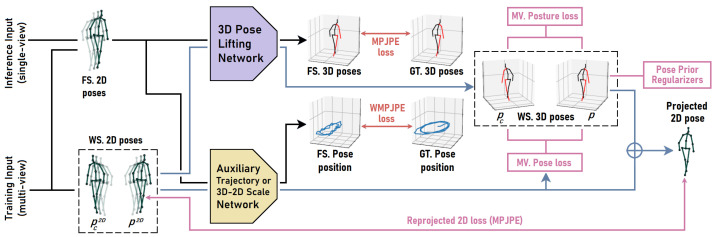
Our semi-supervised scheme for 3D human pose estimation consists of a fully supervised (FS) pipeline (in black) and a weakly supervised (WS) pipeline (in blue). Each training batch contains three subsets of 2D pose inputs. The 1st subset of 2D poses passes through the fully supervised pipeline, which estimates their 3D poses and positions and minimizes the supervised losses (in red) with ground truths (GTs). The other subsets of 2D pose inputs contain 2D poses $p^{2D}$ and matching 2D poses from other camera viewpoints $p_{c}^{2D}$. These 2D poses are fed into the weakly supervised pipeline to estimate their 3D poses ($p,p_{c}$) and positions. The 2nd subset of estimated 3D poses *p* and corresponding positions are combined to project 2D poses and minimize the reprojected 2D loss. Pose prior regularizers [6] are also enforced on *p*. Our proposed multi-view (MV) pose and posture losses are optimized between *p* and $p_{c}$. We train an instance of the VPose3D [41] 3D pose lifting network.

Our semi-supervised training scheme, illustrated in Figure 4, adopts the dual-branch (fully and weakly supervised) pipeline proposed by Pavllo et al. [41] to train a 3D pose model and an auxiliary pose trajectory model.

We regularize the optimization of the weakly supervised 2D reprojection loss using the biomechanical pose prior regularizers proposed by Amadi and Agam [6]. Each training batch is made up of 3 parts: (1) A set of 2D poses (with corresponding 3D pose annotations) for the fully supervised branch; (2) a set of 2D poses for the weakly supervised branch; and (3) a matching set of 2D poses from other camera viewpoints corresponding to the second set of 2D poses, also for the weakly supervised branch. Hence, for a batch of *k* fully supervised 2D poses, we append m·k 2D poses, where *m* − 1 is the number of additional camera viewpoints selected per weakly supervised pose. This setup allows us to optimize multi-view pose and posture consistency during training but maintain monocular 3D pose estimation at inference. We minimize a generic multi-view pose consistency loss $L_{\mathrm{pose}}^{{}^{\prime }}$ in the weakly supervised branch when camera extrinsic parameters are obtainable. The trajectory model estimates the 3D position of the pose $t^{c}$ with respect to the observing camera. Combined with the camera’s extrinsic parameters $Q^{c}$, we transform the estimated pose $p^{c}$ (in camera frame *c*) to a 3D pose in world coordinates $p^{{}^{\prime }c}$.

(8) $$\begin{array}{cc} p^{{}^{\prime }c} & =T(p^{c}+t^{c},Q^{c})\\ L_{\mathrm{pose}}^{{}^{\prime }} & =\frac{1}{m|P||J|}\sum_{i=0}^{\left|P\right|}\sum_{j=0}^{\left|J\right|}\sum_{c=1}^{m}{∥p_{i,j}^{{}^{\prime }0}-p_{i,j}^{{}^{\prime }c}∥}_{2} \end{array}\;$$

The multi-view pose loss is computed in Equation (8) as the mean Euclidean distance between joint pairs ($p_{i,j}^{{}^{\prime }0}$ and $p_{i,j}^{{}^{\prime }c}$) of the corresponding multi-view poses transformed to world coordinates. *P* is the first set of estimated 3D poses in the weakly supervised branch, and *J* is the set of joints in a 3D pose. T denotes the transformation function. Given a pose $p_{i}^{{}^{\prime }0}$∈*P*, $p_{i}^{{}^{\prime }c}$ is a corresponding pose estimated from another viewpoint that is contained in the second set of multi-view poses in the weakly supervised branch.

We apply horizontal flip augmentation to the 2D pose inputs of the weakly supervised branch to generate more unlabeled training data. This simple pose augmentation technique has been effective in previous works. However, once 2D poses are flipped in the image frame, we expect the resulting 3D pose to be flipped in the camera frame. This will cause a mismatch in multi-view 3D poses as each pose is flipped in its camera frame and will not align when transformed to standard world coordinates. Hence, we cannot optimize the multi-view pose consistency loss for such poses even if the camera’s extrinsic properties are known, as this will lead to degenerated results. We can, however, optimize multi-view posture consistency loss for the reflected poses, as the posture remains consistent across viewpoints even after horizontal flip augmentation.

#### 3.3.2. Semi-Supervision without Camera Parameters

Although most 3D pose datasets provide camera parameter annotations, we understand that camera parameters are not so easily obtainable for crowdsourced in-the-wild video data. Since the ultimate goal of semi-supervised pose estimation is to leverage these unlabeled in-the-wild training data, we propose a modified semi-supervised scheme that does not rely on intrinsic and extrinsic camera parameters.

The main objective function of the weakly supervised branch is the reprojected 2D loss, which projects the encoded 3D pose back to the 2D image space and computes the Euclidean distance between keypoints of the input 2D pose and the projected 2D pose. The camera’s intrinsic parameters are necessary to project 3D poses to 2D poses. Hence, the reprojected 2D loss cannot be optimized without the camera’s intrinsic properties. We bypass having to estimate the camera’s internal parameters by replacing the non-linear projection with an orthographic projection. Orthographic projection gives an acceptable approximation of non-linear perspective projection up to scale when images are captured at a short distance and from cameras with negligible skew and distortion effects. It is safe to assume that this is the case for most crowdsourced video data. We then replace the auxiliary trajectory model with an auxiliary scale model that estimates the 3D-to-2D pose scale factor. Note that, as in Section 3.3.1, the 3D poses are always estimated with respect to the root joint. That is, the pelvis joint should be at the Cartesian origin. The orthographic reprojected 2D loss is computed in Equation (9) given an input 2D pose ${\hat{p}}_{i}^{2D}$, estimated 3D pose $p_{i}$, and 3D–2D scale factor $s_{i}$. ${\hat{p}}_{i,r}^{2D}$ is the 2D position of the root joint.

(9) $$\begin{array}{cc} p_{i}^{2D} & =\left(p_{i}^{\left(x,y\right)}\cdot s_{i}\right)+{\hat{p}}_{i,r}^{2D}\\ L_{\mathrm{orth}-2D} & =\frac{1}{\left|P||J\right|}\sum_{i=0}^{\left|P\right|}\sum_{j=0}^{\left|J\right|}{∥p_{i,j}^{2D}-{\hat{p}}_{i,j}^{2D}∥}_{2} \end{array}\;$$

where $p_{i}^{\left(x,y\right)}$ is the orthographic projected 2D pose that excludes the depth of the 3D pose $p_{i}$. In addition, we optimize our proposed multi-view posture consistency loss in Equation (7). Note that multi-view pose consistency loss is not applicable in this scenario because we assume that the camera’s extrinsic parameters are not available. The results show that posture loss significantly boosts the performance of semi-supervised pose estimators trained without camera parameters.

### 3.4. Bone Orientation Error as a Posture Metric

The proposed MPBOE is a notable posture evaluation metric because it captures and isolates errors to the exact bones that are incorrectly oriented. This property is quite unlike the P-MPJPE, a 3D pose protocol that technically measures posture alignment between poses. The rigid alignment procedure of the P-MPJPE computes an optimal rotation matrix, translation vector, and scale factor that best aligns a predicted pose to the ground-truth pose, thereby implicitly assessing posture. However, because an optimal rotation matrix is computed for the entire pose, an error in one joint is shared with other joints. In other words, the significant deviation of a joint is dampened, as it is distributed to other joints. This causes other more accurately predicted joints to further deviate from the ground truth. Therefore, we cannot pinpoint the most faulty joints when analyzing errors per joint. In contrast, because the bone orientation alignment procedure of the MPBOE aligns each bone separately, it can isolate errors to defaulting bones and corresponding quadruplet joints.

**Figure 5:**
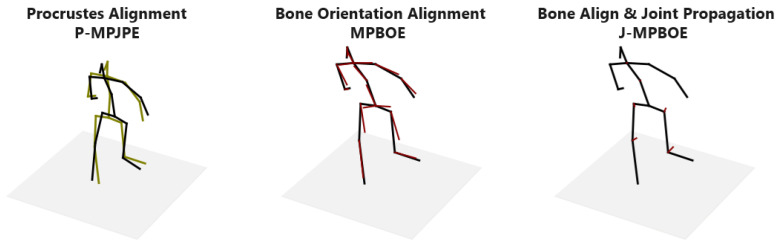
Visualization of posture metrics. Unlike the P-MPJPE, our proposed MPBOE highlights defaulting bones (in red) of the altered pose (in green) that change the posture of the sample pose (in black). Notice in P-MPJPE how most joints are displaced by some error after Procrustes alignment. In contrast, MPBOE shows the orientations of a few bones (e.g., right and left thigh) are off after bone orientation alignment and only a few joints show significant position displacement (illustrated by red line) in J-MPBOE, after propagating bone orientation errors to neighboring joints.

This distinguishing property is illustrated in Figure 5 and demonstrated in detail in Appendix A. To reconstruct the altered pose (in green), a sample 3D pose (in black) is shrunk and translated a distance to the left. We then slightly rotate the upper body at the pelvis joint. The outcome is that the original and altered pose now has a similar posture except for the lower-torso region. Observe that the best fit of the P-MPJPE shows an offset at almost all joints. Whereas, the MPBOE reveals the most significant deviations in the thigh and thorax bones that are in the lower-torso region, while other bones are in near-perfect alignment. In the results section, we show the joint errors of existing protocols compared to our proposed posture metric defined in Equation (6). Note that the distance function *d* can be the cosine similarity, L1-norm, or L2-norm. We compute the L2-norm.

**Proof of Metric Property.**  The MPBOE qualifies as a metric because it satisfies the identity, positivity, symmetry, and triangle inequality properties of a metric space. This follows directly from the property of the distance measure *d*. Given two distinct postures $p_{x}^{{}^{\prime }}$ and $p_{y}^{{}^{\prime }}$ (Free-Bone vectors of 3D poses after bone orientation alignment), notice that $d(p_{x}^{{}^{\prime }},p_{x}^{{}^{\prime }})=0$ and $d\left(p_{x}^{{}^{\prime }},p_{y}^{{}^{\prime }}\right)=d\left(p_{y}^{{}^{\prime }},p_{x}^{{}^{\prime }}\right)$. Given a third posture $p_{z}^{{}^{\prime }}$ that further deviates from $p_{y}^{{}^{\prime }}$, we expect $d\left(p_{x}^{{}^{\prime }},p_{z}^{{}^{\prime }}\right)\leq d\left(p_{x}^{{}^{\prime }},p_{y}^{{}^{\prime }}\right)+d\left(p_{y}^{{}^{\prime }},p_{z}^{{}^{\prime }}\right)$. Proving the positivity property requires slightly more intuition. Given a posture $p_{x}^{{}^{\prime }}$, another posture $p_{y}^{{}^{\prime }}$ can be generated that is very similar to $p_{x}^{{}^{\prime }}$ except that we move the Anchor keypoint of a bone $p_{x,i}^{{}^{\prime }}$ within the Anchor-Plane. Because the Anchor-Plane is unchanged, the orientation of the bone in both postures will align. Hence, $d(p_{x,i}^{{}^{\prime }},p_{y,i}^{{}^{\prime }})=0$, although the posture of the bones relative to their quadruplet keypoints is not the same. However, the deviation of that Anchor keypoint in $p_{y}^{{}^{\prime }}$ will affect the orientation of a neighboring bone $p_{y,j}^{{}^{\prime }}$ when it is used as the Pivot, Axis, or Free keypoint during alignment. Hence, $d(p_{x,j}^{{}^{\prime }},p_{y,j}^{{}^{\prime }})>0$. Therefore, $d(p_{x}^{{}^{\prime }},p_{y}^{{}^{\prime }})>0$. □

### 3.5. Bone Orientation Error Propagated to Joints

The bone orientation error (MPBOE) is bone-centric because it computes the orientation deviation between pairs of aligned bones. However, 3D pose estimation is joint-centric, as we are often interested in joint position errors. The orientation error of each bone can be propagated to the quadruplet joints used to align the bone, resulting in the **joint-propagated mean per-bone orientation error (J-MPBOE)**. We achieve this by attributing a weight $\beta_{i}$ to each quadruplet joint *i* of a bone. The error of a joint is accumulated by computing the weighted sum of the errors of all bones that use the joint (as one of the quadruplet keypoints) during alignment. To clarify, let $T_{j}=\left\{:\left(e_{b},\beta_{i}\right)\right\}$ be the set of tuple pairs of bone errors $e_{b}$ (with joint *j* as a quadruplet keypoint) and corresponding quadruplet keypoint weight $\beta_{i}$. The accumulated error of the joint $e_{j}$ is computed as in Equation (10).

(10) $$e_{j}=\sum_{T_{j}:\left(e_{b},\beta_{i}\right)}\beta_{i}e_{b}\;$$

Provided the weight of all quadruplet keypoints for each bone sums to 1.0, the bone orientation error will be properly dispersed to affected joints without increasing or decreasing the cumulative posture error. We set the weights for the Free, Pivot, Axis, and Anchor keypoints to 0.95,0.03,0.01, and 0.01 for all bones, effectively assigning more importance to the Free and Pivot keypoints that define the bone. Therefore, the J-MPBOE captures and concentrates 3D pose reconstruction errors to the exact out-of-position joints that cause incorrect posture and bone orientations.

## 4. Experiments and Results

We executed various evaluation experiments to answer the following four questions: (1) Can the addition of posture loss alone improve the performance of pose estimators (especially in a semi-supervised setting with estimated 2D poses and without camera parameters)? (2) How much more do semi-supervised pose estimator networks learn from unlabeled data when bootstrapped with multi-view pose and posture loss, and how does this impact pose estimation accuracy? (3) Are the improvements from multi-view pose and posture loss consistent across different datasets? (4) What peculiar characteristics of the proposed posture evaluation metric, if any, can be observed?

### 4.1. Experiment Setup

#### 4.1.1. Training and Inference

The training pipeline was designed to utilize training data examples from two data generators in each iteration. The first data generator fed a batch containing tuples of input 2D poses and their corresponding 3D pose ground truths. Multi-view 2D poses were not compiled by this generator. The training examples from this generator were fed into the fully supervised branch of the semi-supervised network. The second data generator fed a batch of multi-view 2D poses into the weakly supervised branch of the semi-supervised network. Note that this data generator did not produce the 3D pose annotations needed for direct supervision. Rather, by design, our weakly supervised branch utilized the estimated 3D poses from different viewpoints to optimize the network by enforcing multi-view posture and pose consistency and 3D pose biomechanical properties. Sets of multi-view poses from *m* = 4 cameras were selected for weak supervision. Our models were trained with the Adam optimizer for about 200k iterations with the learning rate exponentially decaying from (0.001) to ($1\times 10^{5}$) every 500 steps, a dropout of (0.1), and batch normalization.

At inference, our trained models estimated a 3D pose given 2D pose(s) from a single viewpoint. Unless otherwise stated, we performed estimated 3D pose augmentation during inference. This involved estimating a 3D pose given a 2D pose (or temporal sequence of 2D poses), and another 3D pose was estimated for the horizontally flipped 2D pose(s). Next, we reversed the horizontal flip of the second 3D pose before computing the final 3D pose joint positions as the average of the first and second 3D poses’ joint positions. This inference-time pose augmentation allowed the two network trials to correctly estimate the 3D poses and average the performance. We evaluated the accuracy of 3D pose estimation using the MPJPE, P-MPJPE, MPBOE, and J-MPBOE. Note that we could evaluate the MPJPE on 3D poses estimated by our networks because all configurations of our proposed semi-supervised network reconstructed a 3D pose with respect to the observing camera. This was ensured by the optimization of the MPJPE loss in the fully supervised branch of the semi-supervised framework. In other words, the 3D poses estimated by our models were not orientation- or scale-normalized. All models were trained and evaluated on Nvidia RTX 1080 GPU servers, which handled all computations comfortably.

#### 4.1.2. Datasets and Pose Models

We trained and evaluated the models on the **Human3.6M (H36M)** [1] dataset with video data and 2.1 M annotated poses. Following the convention of previous works [6,25,40,41,52], we conducted training on subjects 1,5,6,7,8 and evaluation on subjects 9,11. The training set was split into a fully supervised subset with 3D pose annotations and a weakly supervised subset with multi-view 2D pose inputs. We trained different models with an increasing number of fully supervised data. We started with 0.1%S1 to S156 as the fully supervised subset. Note that the weakly supervised subset decreased as the fully supervised subset increased. This setup was used in previous works to simulate labeled data scarcity and was intended to test the effectiveness of semi-supervised techniques in high and low labeled-to-unlabeled training data ratios. We also evaluated our models on the **MPI-INF-3DHP (3DHP)** [4] 3D pose dataset with 1.3 M frames. Compared to H36M, 3DHP contains a more diverse collection of 3D poses and movements. We performed cross-validation on 3DHP’s test set to evaluate our models’ ability to generalize to unseen data from a different domain.

We utilized the **VideoPose3D (VPose3D)** human pose estimation network architecture in this study [41]. Thus, we applied the proposed semi-supervised scheme to train the temporal dilated convolution neural network of VPose3D – a monocular 2D–3D pose lifting network capable of lifting a single 2D pose to a 3D pose or a temporal sequence of 2D poses to a 3D pose. We refer to the former as single-frame monocular pose estimation and the latter as temporal monocular pose estimation. We trained and evaluated both types of pose estimation. Therefore, the input to the networks was either a single 2D pose or a temporal sequence of 2D poses. We used **HR-Net** [53] -detected 2D poses to train and evaluate the networks and also determined how the networks performed with ground-truth 2D poses.

### 4.2. Results and Comparisons

The methods presented in this work are intended to utilize estimated 2D poses from pretrained 2D pose detectors. As such, we evaluated the performance with 2D poses detected by a pretrained HR-Net [53] pose detector. We followed a real-world inference setup where estimated 2D poses from pose detectors are used to estimate 3D poses. In addition, we established the performance of our methods when utilizing 2D pose annotations of the dataset for two reasons: (1) to provide a fair comparison with other related works that report performance when using ground-truth 2D poses, and (2) to assess the potency of our method without carrying over the errors of a pretrained 2D pose detector. This allowed for a direct assessment of our 3D pose lifting network independent of the accuracy of the chosen 2D pose detector.

#### 4.2.1. Semi-Supervision on H36M with Full Supervision on S1

To aid direct comparisons with leading semi-supervised pose estimation works, we followed the convention of evaluating our networks’ performance in a limited labeled data scenario where 3D annotations were provided only for the first subject (S1 in the H36M training set) for direct supervision, while pose estimation on the remaining four subjects (S5–8) of the training set were weakly supervised. Table 1 compares the performance of leading semi-supervised, single-frame pose estimators that estimate a 3D pose given a 2D pose or image. Table 2 compares the performance of leading semi-supervised pose estimators that estimate a 3D pose given a temporal sequence of 2D poses or a video clip.

Ours–MvP in Table 1 and Table 2 indicates the version of our semi-supervised network trained with the addition of our multi-view posture loss and with estimated 2D pose inputs. Note that this configuration is fitting for most real-world applications where ground-truth 2D poses and extrinsic camera parameters are unknown. Nevertheless, Ours–MvP outperformed leading single-frame semi-supervised methods that use ground-truth 2D pose inputs (PoseAug) and a combination of 2D pose and image inputs (EpipolarPose and Iqbal et al.). Our model just about outperformed PoseAug, which uses a generative adversarial network (trained on ground-truth 2D and 3D poses of the first subject) to augment poses in the training set, thereby generating more 2D and 3D pose pairs to fully supervise the network. Unlike EpipolarPose, Ours–MvP did not use GT 2D poses. The addition of multi-view pose consistency loss (Ours–MvP&P) further improved the model accuracy to a 54 mm MPJPE. This setup required camera extrinsic parameters, which are obtainable in some real-world applications.

We compared our models’ performance to that of leading temporal semi-supervised 3D pose estimators that reconstruct a 3D pose from a temporal sequence of 27 2D poses or 27 video frames. These methods leverage temporal information to estimate more accurate 3D poses than single-frame pose estimators. The performance of Ours–MvP in Table 1 and Table 2 shows that the accuracy boost from temporal cues was only about 1 mm in the MPJPE, P-MPJPE, and J-MPBOE. This suggests that the posture learning cues from multiple viewpoints that were distilled by our proposed multi-view posture consistency loss in single-frame pose estimation significantly compensated for the absence of temporal information. The addition of pose consistency loss to our temporal semi-supervised framework (Ours–MvP&P) decreased the pose estimation error by an additional 2.6 mm in the MPJPE. Observe that the accuracy of Ours–MvP&P was close to that of the methods proposed by Pavllo et al. and Amadi and Agam, even though their pose estimators were trained and tested with ground-truth 2D pose inputs. We achieved comparable results with less accurate 2D poses estimated by a pretrained HR-Net pose detector.

When we trained and tested our models with ground-truth 2D poses (following Pavllo et al. and Amadi et al.), we recorded an additional 10 mm MPJPE decrease in the pose estimation error (comparing Ours–MvP&P 🟉 and Ours–MvP&P). This showed that our semi-supervised framework was superior to those of Amadi and Agam, and Pavllo et al. Note that just like our method, the semi-supervised frameworks proposed by Pavllo et al. and Amadi et al. were a combination of a fully supervised branch and a weakly supervised branch with 2D reprojected loss as the main objective function of the weakly supervised branch. Our framework differed from theirs in that Pavllo et al. introduced a secondary mean bone length error loss term to the weakly supervised branch, while Amadi and Agam enforced biomechanical pose regularization constraints on the weakly supervised branch. Neither framework leveraged multi-view information to train a more robust monocular 3D pose estimator.

Our model trained with multi-view pose and posture loss (Ours–MvP&P 🟉) also outperformed AdaptPose (42.2 vs. 42.5 MPJPE, 31.8 vs. 34.0 P-MPJPE). Observe that the 3D posture accuracy (assessed by P-MPJPE) of the 3D poses estimated by our method was better than that of AdaptPose. It is important to note that both methods were fundamentally different in how they approached semi-supervised pose estimation, although they used the same information in different ways. The motion GAN of the AdaptPose model reported in Table 2 was trained on ground-truth 2D and 3D poses of S1 and ground-truth 2D poses from video data of the remaining subjects (S5–8). The optimized human motion generator (HMG) then generated additional synthetic 2D–3D pairs for training the 3D pose lifting network in full supervision. At inference, the AdaptPose model predicted a 3D pose given GT 2D poses of a video clip input. In contrast, Ours–MvP&P 🟉 was trained with ground-truth 2D pose inputs from different viewpoints. We did not synthesize 2D–3D pose motion data to directly supervise the 3D pose lifting network. Instead, we leveraged multi-view posture and pose analysis to loosely supervise non-trivial 3D pose estimation for subjects 5–8 without 3D pose annotations. Note that we did not use additional training data. We simply curated each training batch to contain sets of multi-view poses already existing in the dataset. At inference, our model predicted a 3D pose given a temporal sequence of ground-truth 2D poses from a single viewpoint. Both methods used intrinsic and extrinsic camera parameters during training, although AdaptPose did not require camera parameters for subjects 5–8. We recognize that the inference setting with ground-truth 2D pose inputs is not ideal. We applied this setting to compare our results with previous semi-supervised learning methods that have reported results with 2D GT inputs. Nevertheless, we speculate that our method could achieve comparable performance with improved 2D pose detection, which is expected as SOTA 2D pose estimation improves. Note that all methods in Table 2 used VPose3D as the baseline 2D–3D pose lifting network.

#### 4.2.2. Ablation of Camera Parameters with Increasing Full Supervision

**Figure 6:**
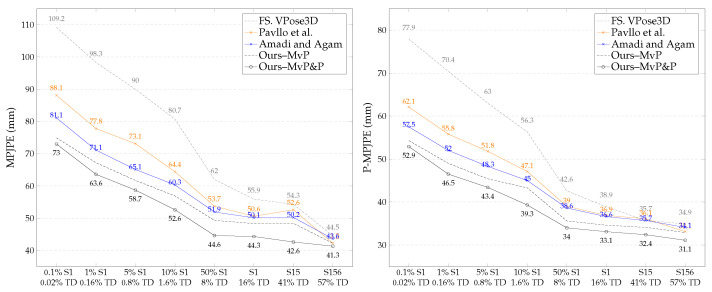
Comparing SOTA semi-supervised frameworks used to train VPose3D backbone with our proposed semi-supervised scheme bootstrapped with pose regularizers, multi-view posture loss (Mv-P), and multi-view pose loss (Mv-P&P). Our models consistently outperformed leading methods on all configurations of increasing subsets of fully supervised training data. FS indicates the baseline VPose3D network trained with the fully supervised pipeline only. Camera parameters were provided during training; FS. VPose3D [41]; Pavllo et al. [41]; Amadi and Agam [6].

**Figure 7:**
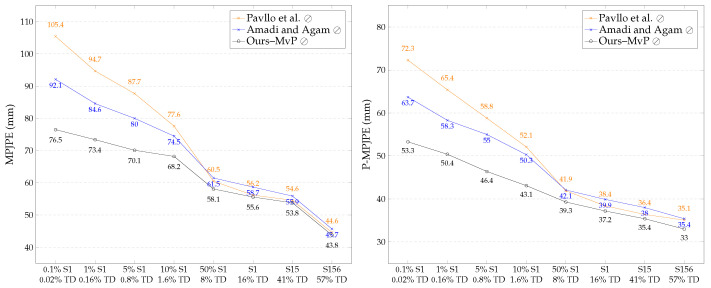
Corresponding semi-supervised VPose3D lifting network trained without camera parameters (denoted by ⊘). Observe the error reduction by our proposed semi-supervised framework with multi-view posture loss (Mv-P) compared to leading methods; Pavllo et al. [41]; Amadi and Agam [6].

To evaluate the significance of our proposed pose and posture consistency loss terms in a limited-data scenario, we trained VPose3D on increasing fully supervised subsets. We started with 0.1%S1 (containing 0.02% of H36M the training data) to S156 (poses from subjects 1, 5, and 6 containing 57% of the H36M training data). We trained the baseline VPose3D lifting network using the semi-supervised learning frameworks proposed by Pavllo et al. and Amadi et al. and our proposed pipelines (Ours–MvP and Ours–MvP&P). Figure 6 shows the results of the models trained with camera parameters, and Figure 7 contrasts this to the performance of the same models trained without camera parameters. Note that the X-axis of Figure 6 and Figure 7 denotes the source of the 2D and 3D pose ground truths used for full supervision. For example, “50% S1 – 8% TD” implies that 50% of the poses from the first subject were fully supervised, while the remaining poses from subjects 5–8 were weakly supervised. This would be equivalent to 8% of the entire training data used in full supervision. Observe that both our proposed semi-supervised frameworks trained with and without camera parameters consistently outperformed the leading methods in each subset. Our pose estimator trained with camera parameters, pose prior regularizers, and multi-view pose and posture consistency loss (Ours–MvP&P) achieved SOTA results, decreasing the MPJPE by −6.6 mm (11%) on average. The highest percentage decrease of −7.3 mm in the MPJPE (14%) was observed for 50%S1.

The results also show that our novel multi-view posture loss (Ours–MvP) was effective as a standalone multi-view consistency loss in the weakly supervised branch, particularly when training without intrinsic or extrinsic camera parameters, as in Ours–MvP ⊘. Note that in the weakly supervised branch of all methods, we replaced the non-linear reprojected 2D loss, which required intrinsic camera parameters, with our proposed orthographic reprojected 2D loss ($L_{\mathrm{orth}-2D}$). The addition of multi-view posture loss to the semi-supervised framework without camera parameters further decreased the MPJPE by −6.1 mm (8%) on average. We observed that the effect of our proposed posture loss was more prominent in scarce-supervised-data scenarios, where fewer than 2% of the training data were fully supervised (i.e., 0.1%S1 to 10%S1). We recorded a substantial −15.6 mm (17%) decrease in the MPJPE and −10.4 mm (16%) decrease in the P-MPJPE for 0.1%S1. With just multi-view posture consistency loss as the supporting loss term in the weakly supervised branch, our models were still capable of predicting accurate non-trivial 3D poses. This shows the significance of 3D posture correctness in 3D pose estimation when posture loss is optimized.

#### 4.2.3. Ablation of Supervision with Little to No 3D Pose Annotations

There have been several promising works on weakly, self-, and unsupervised 3D human pose estimation in recent years. These works have proposed learning schemes that do not rely on 3D pose annotations or paired image-to-3D or 2D-to-3D pose supervision. Although our work proposes a semi-supervised scheme, we compared a specific case where 0.1% of the poses of S1 were fully supervised and the poses of the other four subjects (S5–8) were weakly supervised. This resulted in only 244 fully supervised training examples with 3D pose annotations and over 1.3 M weakly supervised training examples without 3D pose annotations. To offset the benefits of full supervision, we did not train on the remaining 99.9% of poses from S1, effectively discarding over 248 k training examples. In contrast, the weakly/self-/unsupervised methods in Table 3 were trained on all 1.5 M poses of the H36M training set (poses from S1 and S5–8). Table 3 shows the superiority of our pose models to other weakly supervised methods when trained with very little 2D-to-3D paired supervision on 0.02% of the training data. The accuracy of our single-frame model trained with 2D GT poses (Ours–MvP&P 🟉) matched that of the single-frame TriPose model. Like our framework, TriPose [38] is a monocular weakly-supervised training scheme that leverages multi-view 2D poses during training. Unlike our framework, TriPose estimates relative camera orientations, which are combined with input 2D poses from multiple views to triangulate a 3D pose. The triangulated 3D pose is then used as pseudo-annotations to supervise the 2D-to-3D lifting network. Our temporal model trained with HR-Net-detected 2D poses (Ours–MvP&P) significantly outperformed the TriPose temporal pose estimator trained with AlphaPose-estimated 2D poses, achieving a 10% reduction in the MPJPE (−6.1 mm). Note that RepNet estimates trivial oriented 3D poses—hence the lower reported MPJPE and P-MPJPE values.

#### 4.2.4. Cross-Dataset Evaluation on 3DHP

**Figure 8:**
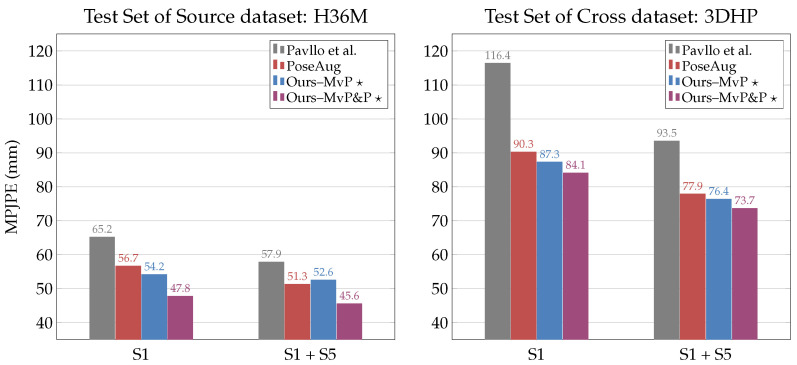
Cross-dataset evaluation (in MPJPE) of single-frame VPose3D network trained on poses from H36M subjects (S1 and S1+S5) and evaluated on the test sets of H36M (**left**) and 3DHP (**right**). Our models trained VPose3D with our proposed semi-supervised scheme, that is, full supervision on the indicated subject(s) (S1 or S1 + S5) and weak supervision on the remaining subjects of the H36M training set (i.e., S5–8 or S6–8); Pavllo et al. [41]; PoseAug [25].

To test how well our semi-supervised models generalized to unseen data from a different domain, we trained the backbone VPose3D network on the training subset of H36M and evaluated its performance on the test set of both H36M and 3DHP. We directly compared our semi-supervised models to the baseline VPose3D network proposed by Pavllo et al. and those of PoseAug [25], which were evaluated in the same setup. Note that our networks were trained with our proposed semi-supervised learning scheme, that is, with full supervision on S1 and S1 + S5 and weak supervision on S5–8 and S6–8. In contrast, the baseline and PoseAug models were fully supervised on S1 and S1 + S5, and PoseAug generated additional 2D–3D paired poses from the training subset to supervise its 3D pose lifting network. Figure 8 shows that the superior performance of our methods on H36M carried over to 3DHP. Our semi-supervised framework with multi-view posture loss (Ours–MvP 🟉) significantly reduced the 3DHP pose estimation error of the baseline network by 25% (−29.1 mm MPJPE) and 18% (−17.1 mm MPJPE) when trained with fully supervised subsets of S1 and S1 + S5, respectively. This shows that our models can learn robust features that generalize well to unseen poses from a different domain. Compared to PoseAug, our models trained with multi-view pose and posture loss further decreased the 3DHP pose estimation error by 7% (−6.2 mm MPJPE) and 5% (−4.2 mm MPJPE) for S1 and S1 + S5, respectively. We also noticed that the effect of our semi-supervised framework was reduced as the weakly supervised subset decreased. This behavior was consistent for both the H36M and 3DHP datasets and our observations in Figure 6 and Figure 7. We can say that the resulting pose estimation accuracy of our proposed semi-supervised learning scheme was directly proportional to the amount of unlabeled data used in weak supervision.

#### 4.2.5. 3D Posture Protocol Assessment

**Figure 9:**
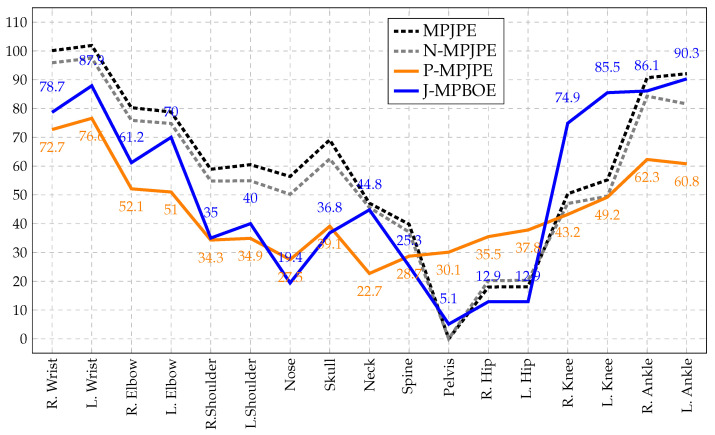
Per-joint assessment of 3D pose (MPJPE and N-MPJPE) and posture (P-MPJPE and J-MPBOE) evaluation protocols. Results were derived from evaluating our semi-supervised VPose3D network (with full supervision on 10%S1) on the H36M test set with inference-time augmentation. We computed the mean error of all poses per joint. Observe that of the two posture protocols, J-MPBOE was better at concentrating errors on hard-to-estimate joints.

We show the per-joint pose and posture errors of our 3D pose model evaluated on the H36M test set in Figure 9 to highlight the unique properties of our proposed posture evaluation metrics. The MPJPE and scale-normalized MPJPE (N-MPJPE) are established 3D pose evaluation protocols, whereas the P-MPJPE and proposed MPBOE and J-MPBOE are evaluation protocols that assess the 3D posture quality of a 3D pose. Recall that the J-MPBOE is derived by propagating the bone orientation error of a bone to its culprit, neighboring joints. In a sense, the J-MPBOE is an interpretation of the MPBOE at the joint level. Hence, we present the J-MPBOE for easier comparison with the other joint-based protocols in Figure 9. We focus primarily on the differences between the established P-MPJPE and our proposed J-MPBOE.

Observe that the errors of the J-MPBOE were much more concentrated in the limb joints (e.g., wrists, elbows, knees, and ankles), which are more volatile in 3D poses because of their higher freedom of movement compared to torso joints (e.g., hips, pelvis, and spine). Therefore, they are much more challenging to estimate. In contrast, the P-MPJPE tended to spread the posture error across all joints, effectively diluting the concentration of errors on the joints that were harder to estimate correctly. This highlights an advantage of the MPBOE and J-MPBOE over the P-MPJPE, which is that the MPBOE and J-MPBOE do not disperse 3D posture errors from incorrectly oriented bones or incorrectly positioned joints. Rather, they retain the concentration of errors on culprit bones and joints while assessing overall posture alignment accuracy. Notice that the pattern of the J-MPBOE was more like that of the MPJPE, which does not disperse 3D pose estimation errors. The mean per-joint errors of the J-MPBOE varied, with a range of 85.2 mm and a standard deviation of 30, compared to 53.8 mm and 16 for the P-MPJPE and 101.8 mm and 29.6 for the MPJPE, respectively. The corresponding statistics for the MPBOE (not shown in Figure 9) were a range of 85.6 mm and a standard deviation of 31.1. This property of the MPBOE and J-MPBOE is favorable for the granular assessment of posture quality.

Also, observe that the J-MPBOE emphasized the network’s bias to the right side of the body. Comparing the J-MPBOE of the right and left wrists, right and left elbows, right and left shoulders, and so on, we noticed that the errors of the right joints were significantly smaller than their left counterparts. None of the other protocols highlighted this bias to the same degree as the J-MPBOE, although the MPJPE showed traces of it. We observed the same pattern in the MPBOE. To further investigate this observation, we horizontally flipped the 2D input poses and 3D ground-truth poses of the H36M test set and reran inference. This time, we observed a significant bias to the left body parts. Considering that the bias moved from the right to the left side after flipping the poses, we could rule out our initial suspicion that the bias originated from the network. This suggested that perhaps there is a right-side bias in the 3D pose annotations of the H36M test set.

## 5. Conclusions

We presented a semi-supervised scheme for training 3D pose estimators with few labeled data. Our proposed framework includes our novel posture loss and multi-view pose consistency loss which enable the weak supervision of poses captured from different viewpoints. We presented two variants of our semi-supervised framework—one for training pose models with camera parameters and the other for training without camera parameters. The results showed that both frameworks are very effective at boosting the performance of a pose model trained with many unlabeled data. However, we obtained optimal performance when our semi-supervised pose networks were optimized with the non-linear perspective reprojected 2D loss, biomechanical pose prior regularizers, and the proposed multi-view pose and posture loss terms that leverage available camera parameters. The effectiveness of our posture loss was more notable in the second framework when camera parameters were withheld and the network was trained with an orthographic reprojected 2D loss and multi-view posture loss. The ablation studies and experiments showed that our proposed multi-view pose and posture loss consistently improved the accuracy of the backbone pose estimation network evaluated on different datasets. The semi-supervised frameworks proposed in this paper offer a solution to the problem of limited labeled 3D pose training examples for 3D human pose estimation, as they effectively leverage unlabeled data to train more accurate pose estimators.

We also proposed novel posture evaluation metrics that have the unique property of concentrating 3D posture reconstruction errors on incorrectly oriented bones and incorrectly positioned joints, irrespective of the overall 3D pose orientation. This standout attribute of the MPBOE and J-MPBOE makes them quite unlike the P-MPJPE, which distributes 3D posture reconstruction errors fairly equally among all the joints in a 3D pose. Thus, it is difficult to pinpoint the most critical out-of-position joints that cause the posture of an estimated 3D pose to deviate from the expected posture of its ground-truth 3D pose. Our proposed posture metrics isolate errors to incorrectly estimated bones and joints much better than existing evaluation protocols, making them the posture evaluation protocol of choice for the granular assessment of 3D posture correctness. Our source code implementation of the methods proposed in this work will be made available at github.com/lawrenceamadi/PoseReg.

## Figures and Tables

**Table 1:** SOTA semi-supervised, single-frame 3D pose estimation methods. These include methods that use GAN pose augmentation (AG), leverage multi-view information (MV), and process additional image data to estimate 3D poses (IM). In the 2D column, FT indicates methods that use 2D poses from a pretrained pose detector fine-tuned on the H36M dataset. HR indicates methods that use 2D poses from a pretrained HR-Net pose detector. † and GT denote methods that use ground-truth 2D keypoints only during training and in both training and inference, respectively. We use 🟉 to denote our models trained with ground-truth 2D poses.

Semi-Supervision with FS on S1 (16% of TD) and WS on S5–8							
**Methods**	**AG**	**IM**	**2D**	**MV**	**MPJPE**↓	**P-MPJPE**↓	**J-MPBOE**↓
EpipolarPose [39] CVPR’19		🗸	FT	🗸	65.3	57.2	-
Iqbal et al. [36] CVPR’20		🗸	†	🗸	62.8	51.4	-
PoseAug [25] CVPR’21	🗸		GT		56.7	42.3	-
Amadi and Agam [6] ICIP’22			GT		52.6	37.3	40.6
Ours–MvP 🟉			GT	🗸	48.4	34.3	37.8
Ours–MvP&P 🟉			GT	🗸	43.5	32.7	37.2
Ours–MvP			HR	🗸	56.1	42.2	49.2
Ours–MvP&P			HR	🗸	54.0	41.5	49.1

**Table 2:** SOTA semi-supervised, temporal, monocular 3D pose estimation methods with 27 temporal frames. These include methods that generate synthetic poses (AG), leverage multi-view information (MV), and process video data (IM). In the 2D column, HR indicates methods that use 2D poses from a pretrained HR-Net pose detector, while GT denotes models that use ground-truth 2D poses in both training and inference. We use 🟉 to denote our models trained with ground-truth 2D poses.

Semi-Supervision with FS on S1 (16% of TD) and WS on S5–8							
**Methods**	**AG**	**IM**	**2D**	**MV**	**MPJPE**↓	**P-MPJPE**↓	**J-MPBOE**↓
Pavllo et al. [41] CVPR’19			GT		49.7	36.7	-
AdaptPose [26] CVPR’21	🗸	🗸	GT		42.5	34.0	-
Amadi and Agam [6] ICIP’22			GT		50.1	36.8	40.3
Ours–MvP 🟉			GT	🗸	47.0	33.3	37.2
Ours–MvP&P 🟉			GT	🗸	42.2	31.8	36.7
Ours–MvP			HR	🗸	55.0	41.1	48.2
Ours–MvP&P			HR	🗸	52.4	39.7	47.5

**Table 3:** Pose and posture errors of SOTA weakly supervised methods on H36M. GT denotes methods that use 2D pose ground truths for training and inference, while † denotes methods that use GT 2D poses only during training. FT denotes methods that use a pretrained 2D detector fine-tuned on H36M. AP and HR denote methods that use AlphaPose [54] and HR-Net [53] 2D poses. The NF column represents the number of frames: 1 for a single frame, and 27 for a temporal sequence of 27 2D pose inputs. IM indicates methods that leverage additional image or video data to estimate 3D poses. MV indicates methods that use multi-view information during training. ‡ indicates the models that are trained with extra data. 🟉 denotes our models trained with ground-truth 2D poses.

Unpaired 2D–3D Supervision or Weakly/Self-/Unsupervised Methods on S15–8							
**Method**	**NF**	**IM**	**2D**	**MV**	**MPJPE**↓	**P-MPJPE**↓	**J-MPBOE**↓
Tung et al. [28] ICCV’17	2		GT		79.0	-	-
Zhou et al. [33] ICCV’17	1	🗸	†		64.9	-	-
Dabral et al. [47] ECCV’18 ‡	20	🗸	†		52.1	36.3	-
Wang et al. [44] ICCV’19 ‡	1	🗸	†		83.0	57.5	-
RepNet [32] CVPR’19	1		GT		50.9	38.2	-
EpipolarPose [39] CVPR’19	1	🗸	†	🗸	55.1	47.9	-
EpipolarPose [39] CVPR’19	1	🗸	FT	🗸	76.6	67.5	-
Iqbal et al. [36] CVPR’20	1	🗸	†	🗸	69.1	55.9	-
TriPose [38] CoRR’21	1		GT	🗸	56.7	43.8	-
TriPose [38] CoRR’21	27		AP	🗸	62.9	47.0	-
CanonPose [37] CVPR’21	1		AP	🗸	74.3	53.0	-
ElePose [55] CVPR’21	1		GT		64.0	36.7	-
Ours–MvP&P	1		HR	🗸	59.7	46.2	53.4
Ours–MvP&P 🟉	1		GT	🗸	52.2	39.6	46.0
Ours–MvP&P	27		HR	🗸	56.8	43.8	51.7
Ours–MvP&P 🟉	27		GT	🗸	48.6	37.2	44.4

**Figure A1:**
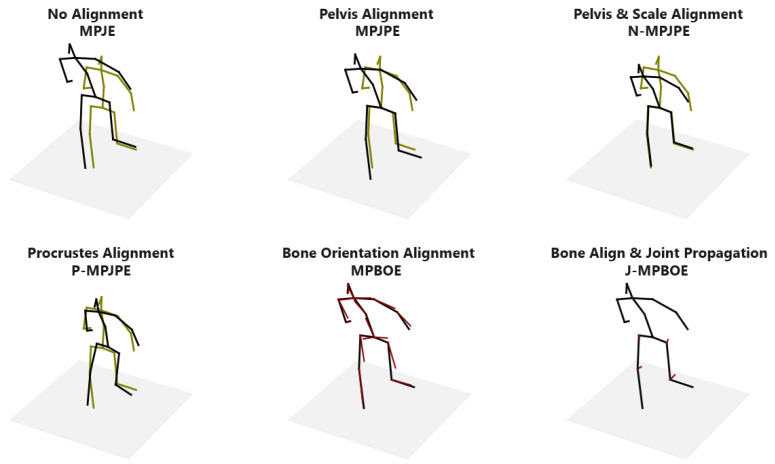
A visual representation of 3D pose and posture reconstruction error protocols. The black figure is the target 3D pose. For illustration, we constructed an altered 3D pose (in green) by enlarging the target pose, translating it, and slightly rotating the upper body at the pelvis joint to change the posture. Observe that, unlike the P-MPJPE, our proposed MPBOE and J-MPBOE highlight the defaulting bones and joints (in red) in the altered pose that cause the change in posture.

**Figure A2:**
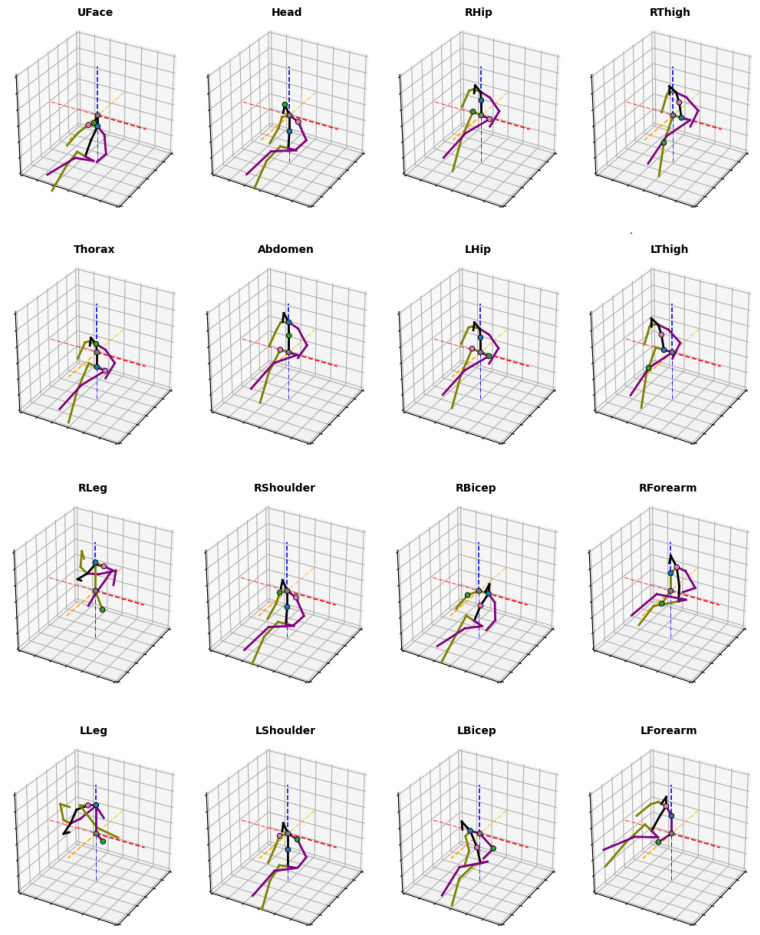
Visualization of the 3D pose transformation effect of our novel bone orientation alignment procedure for each bone. The X-, Y-, and Z-axis are represented by the red, blue, and orange dotted lines, respectively. The Pivot, Axis, Anchor, and Free keypoints are denoted by the gray, blue, pink, and green circles, respectively. The right-side, central, and left-side body parts are color-coded in lemon green, black, and purple, respectively.

## Data Availability

The H36M dataset used in this study can be obtained at the official website vision.imar.ro/human3.6m/description.php (accessed on 20 February 2019). The 3DHP dataset used in this study can be obtained at the official website vcai.mpi-inf.mpg.de/3dhp-dataset/ (accessed on 12 May 2022).

## Abbreviations

The following abbreviations are used in this manuscript:

HPE	Human pose estimation
3D-HPE	Three-dimensional human pose estimation
GAN	Generative adversarial network
PDF	Probability density function
MPJPE	Mean per-joint position error
N-MPJPE	Scale-normalized mean per-joint position error
P-MPJPE	Procrustes-aligned mean per-joint position error
MPBOE	Mean per-bone orientation error
J-MPBOE	Joint-propagated mean per-bone orientation error
H36M	Human3.6M 3D Pose Dataset
3DHP	MPII 3D Human Pose Estimation Dataset
VPose3D	VideoPose3D Pose Estimation Network
HR-Net	High-Resolution 2D Pose Estimation Network

## Appendix A. Error Concentration Property of MPBOE and J-MPBOE Illustrated

To demonstrate MPBOE and J-MPBOE concentration of posture errors compared to P-MPJPE dispersion, we refer to the same example poses as Figure 5 in Section 3. Figure A1 contains a target 3D pose (in black) and an altered 3D pose (in green) that is derived from shrinking and translating the target pose. Then, the upper body joints are slightly rotated about the pelvis joint. Notice that the target and altered pose have similar postures except for the relative orientation between the upper and lower body. The target pose has a slightly bent posture, while the altered pose has an upright posture. The illustrations of Figure A1 visualize the relative positioning of the altered and target pose without alignment (MPJE), after pelvis alignment (MPJPE) and scale normalization (N-MPJPE). 3D pose scale, position, and orientation alignment are done for P-MPJPE, and our proposed bone orientation alignment was performed for the MPBOE and J-MPBOE. The resulting reconstruction errors for each joint and bone are detailed in Table A1.

**Table A1:** The resulting 3D pose and posture reconstruction errors (in millimeters) between target and altered example poses of Figure A1. Notice that the errors are sparse for most joints and bones of the J-MPBOE and MPBOE but concentrated at the few out-of-position joints and bones.

Protocol	R.Wrist	L.Wrist	R.Elbow	L.Elbow	R.Shoulder	L.Shoulder	Nose	Skull	Neck	Spine	Pelvis	R.Hip	L.Hip	R.Knee	L.Knee	R.Ankle	L.Ankle
MPJPE	0.19	0.23	0.20	0.24	0.28	0.27	0.31	0.34	0.27	0.20	0.15	0.17	0.13	0.16	0.13	0.20	0.23
N-MPJPE	0.13	0.20	0.10	0.20	0.18	0.20	0.23	0.25	0.20	0.14	0.11	0.10	0.12	0.13	0.14	0.15	0.15
P-MPJPE	0.03	0.08	0.05	0.05	0.04	0.03	0.06	0.06	0.03	0.02	0.07	0.08	0.06	0.04	0.02	0.08	0.08
J-MPBOE	0.00	0.00	0.00	0.00	0.00	0.00	0.00	0.00	0.00	0.01	0.00	0.02	0.03	0.08	0.11	0.00	0.00
Protocol	R.Radius	L.Radius	R.Humerus	L.Humerus	R.Clavicle	L.Clavicle	Face	Head	Thoracic	Lumbar	R.Waist	L.Waist	R.Femur	L.Femur	R.Tibia	L.Tibia	
MPBOE	0.00	0.00	0.00	0.00	0.00	0.00	0.00	0.00	0.00	0.00	0.00	0.00	0.09	0.11	0.00	0.00	

Observe that the MPBOE correctly registered the most errors in the orientation of the thigh (femur) bones that changed the posture, whereas the P-MPJPE dispersed the errors that should have been concentrated in the torso region (i.e., bones and joints closest to the pelvis) to all the joints. Similarly, for the J-MPBOE, the errors were sparse for joints except those close to the pelvis keypoint that captured the change in posture.

## Appendix B. Configuration of Bone Orientation Alignment Procedure

Here, we reveal important implementation details of our proposed bone orientation alignment procedure in Table A2 and show the outcome illustrated by the 3D pose transformation for each bone in Figure A2.

**Table A2:** Free-Bone alignment configuration. This table details the configuration we used to align each bone of a typical 17-keypoint 3D pose skeleton. The notations are consistent with the explanation of our bone alignment procedure discussed in Section 3.2, which involved the careful selection of Pivot, Axis, Anchor, and Free keypoints and the description of how they are computationally combined to align each bone. The notations R. and L. are for Right and Left, respectively.

Bone or	Quadruplet Keypoints								Superscripts	Axis-Bone
**Body Part**	**Free** $\left(j_{f}^{{}^{\prime }}\right)$	**Pivot** $\left(j_{p}^{{}^{\prime }}\right)$	**Axis** $\left(j_{a}^{{}^{\prime }}\right)$	**Anchor** $\left(j_{c}^{{}^{\prime }}\right)$	$\delta_{f}^{a}$	$\delta_{f}^{c}$	${\vec{v}}_{f}^{k}$	${\vec{v}}_{f}^{c}$	(a,c)	**Aligned to**
Face	Nose	Skull	Neck	R. Shoulder	−1	−1	$j_{c}^{{}^{\prime }}\times j_{a}^{{}^{\prime }}$	$j_{a}^{{}^{\prime }}\times {\vec{v}}_{f}^{k}$	(j,i)	Y-Axis
Head	Skull	Neck	Spine	L. Shoulder	−1	1	$j_{a}^{{}^{\prime }}\times j_{c}^{{}^{\prime }}$	${\vec{v}}_{f}^{k}\times j_{a}^{{}^{\prime }}$	(j,i)	Y-Axis
Thoracic Vertebrae	Neck	Spine	Pelvis	L. Hip	−1	1	$j_{a}^{{}^{\prime }}\times j_{c}^{{}^{\prime }}$	${\vec{v}}_{f}^{k}\times j_{a}^{{}^{\prime }}$	(j,i)	Y-Axis
Lumbar Vertebrae	Spine	Pelvis	Neck	R. Hip	1	−1	$j_{a}^{{}^{\prime }}\times j_{c}^{{}^{\prime }}$	${\vec{v}}_{f}^{k}\times j_{a}^{{}^{\prime }}$	(j,i)	Y-Axis
R. Waist	R. Hip	Pelvis	Spine	L. Hip	1	1	$j_{c}^{{}^{\prime }}\times j_{a}^{{}^{\prime }}$	$j_{a}^{{}^{\prime }}\times {\vec{v}}_{f}^{k}$	(j,i)	Y-Axis
L. Waist	L. Hip	Pelvis	Spine	R. Hip	1	−1	$j_{a}^{{}^{\prime }}\times j_{c}^{{}^{\prime }}$	${\vec{v}}_{f}^{k}\times j_{a}^{{}^{\prime }}$	(j,i)	Y-Axis
R. Femur (Thigh)	R. Knee	R. Hip	Pelvis	Spine	1	1	$j_{a}^{{}^{\prime }}\times j_{c}^{{}^{\prime }}$	${\vec{v}}_{f}^{k}\times j_{a}^{{}^{\prime }}$	(i,j)	X-Axis
L. Femur (Thigh)	L. Knee	L. Hip	Pelvis	Spine	−1	1	$j_{c}^{{}^{\prime }}\times j_{a}^{{}^{\prime }}$	$j_{a}^{{}^{\prime }}\times {\vec{v}}_{f}^{k}$	(i,j)	X-Axis
R. Tibia (Foreleg)	R. Ankle	R. Knee	R. Hip	Pelvis	1	1	$j_{c}^{{}^{\prime }}\times j_{a}^{{}^{\prime }}$	$j_{a}^{{}^{\prime }}\times {\vec{v}}_{f}^{k}$	(j,i)	Y-Axis
L. Tibia (Foreleg)	L. Ankle	L. Knee	L. Hip	Pelvis	1	1	$j_{a}^{{}^{\prime }}\times j_{c}^{{}^{\prime }}$	$j_{a}^{{}^{\prime }}\times {\vec{v}}_{f}^{k}$	(j,i)	Y-Axis
R. Clavicle	R. Shoulder	Neck	Spine	L. Shoulder	−1	1	$j_{a}^{{}^{\prime }}\times j_{c}^{{}^{\prime }}$	${\vec{v}}_{f}^{k}\times j_{a}^{{}^{\prime }}$	(j,i)	Y-Axis
L. Clavicle	L. Shoulder	Neck	Spine	R. Shoulder	−1	−1	$j_{c}^{{}^{\prime }}\times j_{a}^{{}^{\prime }}$	$j_{a}^{{}^{\prime }}\times {\vec{v}}_{f}^{k}$	(j,i)	Y-Axis
R. Humerus (Bicep)	R. Elbow	R. Shoulder	Neck	Spine	1	1	$j_{c}^{{}^{\prime }}\times j_{a}^{{}^{\prime }}$	${\vec{v}}_{f}^{k}\times j_{a}^{{}^{\prime }}$	(i,j)	X-Axis
L. Humerus (Bicep)	L. Elbow	L. Shoulder	Neck	Spine	−1	1	$j_{a}^{{}^{\prime }}\times j_{c}^{{}^{\prime }}$	$j_{a}^{{}^{\prime }}\times {\vec{v}}_{f}^{k}$	(i,j)	X-Axis
R. Radius (Forearm)	R. Wrist	R. Elbow	R. Shoulder	Neck	1	1	$j_{c}^{{}^{\prime }}\times j_{a}^{{}^{\prime }}$	$j_{a}^{{}^{\prime }}\times {\vec{v}}_{f}^{k}$	(j,i)	Y-Axis
L. Radius (Forearm)	L. Wrist	L. Elbow	L. Shoulder	Neck	1	−1	$j_{a}^{{}^{\prime }}\times j_{c}^{{}^{\prime }}$	${\vec{v}}_{f}^{k}\times j_{a}^{{}^{\prime }}$	(j,i)	Y-Axis

Illustrating the Outcome of Bone Orientation Alignment
